# Knowledge, Attitude and Practice of Antenatal Exercise among Pregnant Women in Kuala Selangor

**DOI:** 10.21315/mjms-07-2024-493

**Published:** 2025-02-28

**Authors:** Md Yunus Nur Farhana, Abdul Manaf Rosliza, Ismail Suriani, Alagirisamy Parwathi, Jathin Romizan

**Affiliations:** 1 Centre for Physiotherapy Studies, Faculty of Health Sciences, Universiti Teknologi MARA (UiTM) Cawangan Selangor, Kampus Puncak Alam, Selangor, Malaysia; 2 Department of Community Health, Faculty of Medicine and Health Sciences, Universiti Putra Malaysia, Serdang, Selangor; 3 KPJ Damansara Specialist Hospital, Damansara Utama, Petaling Jaya, Selangor, Malaysia

**Keywords:** knowledge, attitude, practice, antenatal exercise, pregnant women

## Abstract

**Background:**

Pregnancy is associated with significant physical, emotional, and psychological changes. To adapt to these changes and ensure a healthy pregnancy, lifestyle interventions such as regular antenatal exercise (ANE) are vital. Hence, adequate knowledge of ANE among pregnant women is essential to promote the uptake of ANE. This study aimed to assess the knowledge, attitude, and practice (KAP) of ANE, and its associated factors among pregnant women in a suburban district.

**Methods:**

A cross-sectional study was conducted among 571 pregnant women receiving antenatal care at primary health clinics in Kuala Selangor. Demographic data and maternal characteristics, as well as the KAP and associated factors of ANE, were obtained using a self-administered questionnaire. Descriptive statistics of the KAP of ANE were generated. Binary and multivariate logistic regressions were used to determine the predictors of ANE among pregnant women.

**Results:**

About half (53.7%) of the study participants reported adequate knowledge while two-thirds (65.5%) had a positive attitude toward exercise. However, only 38.9% displayed a good practice of ANE. KAP of ANE was significantly associated with higher education, pre-pregnancy physical activity, and having received advice on physical activity. Women with positive attitudes were more likely to have good practice of ANE (AOR = 2.2; 95% CI: 1.5–3.2).

**Conclusion:**

In short, this study indicated a moderate level of knowledge but a positive attitude towards ANE among pregnant women. Nevertheless, their actual practice of ANE needs to be improved. Future health education campaigns should focus on empowering women about the uptake and conduct of physical activity during pregnancy.

## Introduction

Antenatal exercises (ANE) have been shown to improve the health status of women, especially in pregnancy. Among the proven benefits of ANE are shorter labour duration and reduced rates of caesarean delivery (1). A higher percentage of caesarean delivery was reported among pregnant women with physical inactivity (45%) compared to those who were physically active (39%) in Poland (2). ANE can also prevent excessive gestational weight gain and glucose intolerance. In addition, ANE can enhance the physical fitness and cardiovascular endurance of the women, subsequently producing a healthier placental environment for the growth and development of the fetus (3). Furthermore, regular ANE can prevent pregnancy-related complications such as bladder and bowel incontinence, back pain, fatigue, abdomen muscle weakness, obesity, and varicose veins (1). Therefore, the American College of Obstetrics and Gynecologists (ACOG) recommends moderate-intensity exercise for approximately 30 minutes most days a week (4) or all pregnant women, regardless of their physical fitness levels. The lack of exercise during pregnancy may lead to poor muscular and cardiac fitness, excessive maternal weight gain with an increased risk of gestational diabetes mellitus (GDM), varicose veins, lower back pain, and poor psychological adjustment (5).

Physical inactivity is the fourth most crucial risk factor of premature mortality worldwide (6). According to a recent systematic review, there is a high rate of low physical activity levels among pregnant women in high-income countries such as North America and North Europe as compared to East and Southeast Asia nations (7). However, in both developing and developed countries, a lack of knowledge and favourable attitudes towards exercise during pregnancy can result in low physical activity levels (8–10). In Malaysia, there is no national-level data on physical activity among pregnant women. However, studies have shown that pregnant women tend to reduce their physical activities as the pregnancy progresses, hence the increased physical inactivity among pregnant women is not surprising (11–12). A recent study in Kuala Lumpur reported that 38.3% of first-trimester pregnant women were physically inactive, with primigravida, education level, and body mass index being the main determinants of physical inactivity (13). Similarly, two-thirds (64.5%) of pregnant women in Seremban, Malaysia were also found to have physical inactivity (14).

Even though exercise can benefit women, not all of them are fully aware of the importance of physical activity during pregnancy. Even though some women might have the right knowledge about exercising while pregnant, not many of them engage in regular physical activity during pregnancy (15). Therefore, it is essential to raise awareness of the benefits of early pregnancy exercise in mitigating pregnancy-related complications due to physical inactivity. Furthermore, by adopting a culture of regular physical activity, women are better equipped to maintain optimal health and thus reduce the risk of complications associated with sedentary lifestyles in pregnancy. Besides benefiting expecting mothers, this proactive approach will also contribute to the general health wellbeing of society. In Malaysia, only knowledge and attitudes about pelvic floor muscle exercises have been assessed among pregnant women (16). Thus, to address the existing literature gaps, this study aims to assess the knowledge, attitude, and practice (KAP) toward ANE and to identify its associated factors. As the first study in Malaysia to determine the KAP of ANE, the findings of this study may provide vital insight for healthcare providers (HCPs). Baseline data from this study will aid future planning regarding the appropriate strategies to improve pregnancy through exercise.

## Methods

### Study Sample

A cross-sectional study was conducted at seven primary health clinics in Kuala Selangor district, Selangor, on the west coast of Peninsular Malaysia. Kuala Selangor is a suburban area undergoing rapid development with an increasing population of childbearing age (17).

Based on the two-proportion formula, the sample size was calculated with the proportions of pregnant women with a high practice (0.12) and low practice (0.08) of ANE in a previous study (10), with a 5% marginal error and 95% CI (0.05). After accounting for a 20% dropout rate, the sample size was 571. An estimated 1,500 newly pregnant women were proportionately stratified and assigned to each primary health clinic in Kuala Selangor. The estimated number of new pregnant women in each primary health clinic was calculated based on the average number of registered antenatal women per month in 2021. The number of selected respondents was computed based on the proportion of registered pregnant women in 2021. Systematic random sampling was used to select the pregnant women attending the clinics between December 2021 and March 2022. Every second pregnant woman who met the inclusion criteria was invited to join the study. The inclusion criteria were pregnant mothers aged 18–49 years old who could read and understand the Malay language. Those with medical or obstetric complications resulting in ambulatory disabilities and severe psychological conditions were excluded. Information about the study was conveyed to the pregnant women. Those who agreed to participate in the study would need to sign an informed consent form.

### Data Collection

This study applied a self-administered questionnaire adapted from a previous study in Ethiopia with the questionnaire items producing KAP percentages ranging from 83.6% to 97.5%. The intra-class coefficient was determined to be 0.96 (10). The questionnaire was translated into the Malay language for a better understanding of the local population. It was reviewed and validated by six public health and physiotherapy experts to ensure its content validity. Further amendments were made based on their feedback. The Malay version of the questionnaire recorded a high content validity index (CVI) score (Item-CVI between 0.84 and 1.00 and Scale level-CVI > 0.92). Face validity was carried out among 20 pregnant women in a random health clinic that was not included in the study. The feedback from the respondents was assessed and used to further amend the questionnaire.

The first section of the questionnaire used comprised of socio-demographic, socio-economic, obstetric, and health characteristics of pregnant women. The second section focused on the KAP of ANE. The knowledge domain (benefits and contraindications) of ANE consisted of 20 items. Participants who scored more than or equal to the mean value would be deemed as having adequate knowledge. The attitude domain comprised 12 items; those who scored above or equal to the mean score value was defined as having a positive attitude. The practice domain consisted of six items. Women who performed any ANE at least ≥ three times for 20 minutes each time in a week were considered as having good practice of ANE.

### Data Analysis

Descriptive analysis was performed for continuous data (mean ± standard deviation, SD) and categorical data (frequencies and percentages). Regression analysis was used to adjust confounding factors to determine the crude odds ratio (COR) and adjusted odds ratio (AOR) against the dependent variables. Six independent variables in this study were tested simultaneously using the ENTER method to obtain the AOR and *p*-values. Significant predictors of KAP were confirmed when *p* < 0.05. Multicollinearity was confirmed by checking the standard errors of the most significant variables in the model. There was no multicollinearity between the significant variables and the value inflation factor (VIF) < 5.0 (18). SPSS version 27 was used to perform all analyses.

## Results

### Baseline Characteristics

Table 1 summarises the participant’s demographic, maternal characteristics, and health conditions. A total of 571 pregnant women were approached in the clinics and 553 consented and completed the questionnaire, giving a 95.3% response rate. The participants ranged from 19 to 46 years, with a mean age of 29.9 years and an SD of 5.09 years. In terms of academic level, 274 (49.5%) of them were secondary school graduates, another 271 (49%) were university graduates, and 1.5% had only primary education. Half of the women (50.6%) were unemployed. Among those who were employed, more than three-quarters (*n* = 372, 78.5%) had an income level of ≤ RM3,000, and another 102 (21.5%) had an income level ≥ RM5,000. Most of them (*n* = 302, 54.6%) showed an active physical activity level pre-pregnancy. Only 304 (55.0%) of them received advice on physical activity during pregnancy from their HCPs.

More than half of them (55.5%) were in the third trimester. Among them, 232 (42%) were multiparous, 182 (32.9%) were nulliparous, and 139 (25.1%) were primiparous. Most of them (*n* = 341, 61.6%) did not have any medical problems. However, the most common medical illness was GDM (23.2%), followed by bronchial asthma (4.5%).

### Knowledge of the Benefits and Contraindications of ANE

Table 2 shows the participants’ knowledge of the benefits and contraindications of ANE. Most of them (*n* = 311, 56.2%) heard about ANE before. The mean knowledge score (SD) was 10.1 (5.03), with a range of 0 to 18. Based on the mean score, 256 (46.3%) of them had adequate knowledge about ANE. Further analysis of the items revealed that as high as 80.8% of women knew that ANE could help to deal with labour and delivery pain while another 76.1% knew that ANE can increase energy and stamina during pregnancy.

### Attitude towards ANE

Table 3 shows the participant’s attitude towards ANE. The mean (SD) attitude score was 10.6 (2.77). As high as 82.3% of participants had a positive attitude based on the cut-off value. Most of them (94.4%) agreed that ANE helps to prevent pregnancy complications and 504 (91.1%) believed that ANE is safe to practice. However, more than half of them (58.4%) claimed that they did not have sufficient information about ANE.

### Practice of ANE

More than one-third (38.9%) of the participants reported having a good ANE practice based on the definition. The results revealed that most of them (63.3%) practised walking followed by breathing/relaxation exercises as their ANE. Only 13.2% and 10.1% practised pelvic floor exercises and abdominal exercises ≥ three times per week. Most of them (*n* = 338, 61.1%) had a poor practice of ANE with only a frequency of less than three times a week and less than 20 minutes per session (Table 4).

### Factors Affecting Pregnant Women’s KAP of ANE

Based on the multivariable regression analysis, education level and having received advice on exercise were significantly associated with the knowledge of ANE. Compared to those without formal education, those who completed college and university reported adequate knowledge of exercise during pregnancy (AOR = 0.45; 95% CI: 0.3–0.6). Besides, pregnant women who received advice on exercise were 1.55 more likely to have adequate knowledge than those who did not receive any advice (AOR = 1.55; 95% CI: 1.1–2.2).

In addition, pregnant women who practised exercise pre-pregnancy (AOR = 1.7; 95% CI: 1.1–2.7) and received advice on exercise (AOR = 1.95; 95% CI: 1.2–3.1) were more likely to have a positive attitude towards ANE. As for the practice of ANE, the significant predictors were exercising pre-pregnancy, receiving advice on exercise, and having a positive attitude. Those who performed pre-pregnancy exercise were 2.12 times (AOR = 2.12; 95% CI: 1.4–3.0) more likely to practise ANE. The likelihood of good practice also increased by 1.97 times (AOR = 1.97; 95% CI: 1.3–2.9) among those who received advice to exercise. Besides, pregnant women with positive attitudes were 3.0 times (AOR = 2.98; 95% CI: 1.7–5.2) more likely to have a good practice of ANE.[Table t5-12mjms3201_oa]

## Discussion

Overall, the study found that 46.3% of pregnant women in Kuala Selangor reported an adequate knowledge of ANE, higher than studies conducted in Zambia (14%) (19), Colombo, Sri Lanka (16.3%) (20), and Gondar, Ethiopia (39.5%) (10). Demographics, socio-economic status, and healthcare access may contribute to the differences between studies. In a study conducted among 266 pregnant women in Pakistan using a similar study instrument, 45.1% showed sufficient knowledge about ANE. In contrast, other studies reported a slightly lower rate of good knowledge, for example, in the Tigray region (51%) (21), Bahir Dar City, Ethiopia (55.8%) (22), and Nigeria (51%) (3), likely due to variations in sample size and different population demography.

Regarding attitude toward ANE, about 82.3% of the pregnant women in this study had a positive attitude. This is almost in line with a study among 279 pregnant women in Zambia (19), whereby 93% of participants showed a positive attitude. However, the remaining studies in the literature showed a lower rate of positive attitude, for instance, in the Tigray region (56%) (21), Bahir Dar City (53.3%) (22), and Gondar in Ethiopia (55.3%) (10). Depending on the exposure to the importance of exercise during pregnancy, pregnant women may be able to change their misconceptions about exercise and subsequently adopt a positive mindset and behavioural changes.

As for ANE practice, 38.9% of the study participants had a good practice of ANE, similar to the study in Gondar, Ethiopia (30.9%) (10) but lower than Zambia (67%) (19). This difference could be attributed to the higher educational and occupational backgrounds among the Zambian study participants. In comparison, our study participants had a higher level of education compared to studies in the Tigray region (23.5%) (21) and India (18%) (23), hence likely explaining the lower rate of exercise during pregnancy.

In line with the findings above, educational level was a significant predictor of ANE knowledge in this study. Similar results were reported in studies from Brazil (24), Iraq (8), Saudi Arabia (9), Zambia (19), and Gondar (10). Women with higher education levels will actively seek out information from HCPs. They are also more likely to understand the importance of exercise and hence, perform ANE. To empower all levels of women, educational programmes for physical exercise should use simple languages and easy-to-understand terminologies (13) so that they can easily access healthcare information and take better care of themselves.

Next, a positive association between good knowledge of exercise during pregnancy and pregnant women who received advice on exercise was also observed. Studies in Bahir Dar city (22) and Gondar (10) both supported this finding. Women can improve their knowledge of exercises by accessing information from social media or via HCPs. In this digital age, information dissemination through social media is very helpful in creating awareness (25). This finding emphasises the need for tailored awareness and educational programmes to promote physical exercise as part of healthy lifestyles during pregnancy. In addition, HCPs can disseminate advice about exercise to pregnant women during ANC visits to improve their knowledge about the importance of ANE (26).

When assessing factors affecting pregnant women’s attitude toward ANE, those who practised pre-pregnancy exercise were associated with higher odds of having a positive attitude. This is aligned with the findings from studies conducted in Bahir Dar city (22) and Gondar (10), likely because women who exercise during the pre-pregnancy period are more well-versed with the benefits of exercising, hence more willing to remain active while pregnant. Receiving advice about exercise, whether from HCPs or other sources, was another factor that showed a significant relationship with a positive attitude, as reported in the studies in Bahir Dar city (22) and Gondar (10). Advice about the importance of ANE, particularly from HCPs, can alter the woman’s negative perception or myths about exercise during pregnancy, subsequently leading to positive behaviours. Moreover, practising pre-pregnancy exercise and being advised on exercise were also predictors of the good practice of ANE. This finding is supported by the study in Gondar, Ethiopia (10). Women who practice exercise routinely before pregnancy may continue to exercise during pregnancy as they have experienced the benefits of exercise before this. Similarly, advice about the importance of ANE can change pregnant women’s behaviours, thus catalysing positive behavioural modifications (27). Behavioural changes can be enhanced by fostering an attitude, as shown in the studies conducted in Nigeria (3), Ethiopia (10) and Pakistan (28).

This is the first study in Malaysia on the KAP of ANE among pregnant women. One of the limitations of the study included the use of self-reported questionnaires that could give rise to social desirability bias.

## Conclusion

In summary, to further improve the practice and behaviour of ANE among pregnant women in Kuala Selangor, educational and awareness programmes must be implemented to improve their knowledge of ANE. Customised interventions should be developed for pregnant women with different levels of education to cultivate a positive attitude. Pre-pregnancy exercise and advice about ANE should also be emphasised in any related health promotion campaigns. HCPs should aim to provide a comprehensive health education programme that can empower women with ANE-related issues.

## Figures and Tables

**Table 1:** Participant’s socio-demographic, socio-economic and maternal and health characteristics (*n* = 553)

Variable	Number (*n*)	Frequency (%)
Age (years old)		
< 25	78	14.1
25–35	361	65.3
> 35	114	20.6

Race		
Malay	498	90.1
Chinese	11	2.0
Indian	41	7.4
Others	3	0.5

Educational level		
Primary	7	1.5
Secondary	272	49.2
Tertiary	274	49.5
Work status		
Not working	272	49.2
Working	275	49.7
Student	6	1.1

Average household income		
Respond	474	85.7
Not respond	79	14.3
< RM3,000	246	15.9
≥RM3,000	126	26.6
≤RM5,000	87	18.4
RM10,000–RM15,000	15	3.2

Parity		
No children	185	33.5
1–2 children	253	45.8
> 3 children	115	20.8

Gravida		
Primigravida	182	32.9
Multigravida	347	62.7
Grand multigravida	24	4.3

Gestational period		
1st trimester	72	13.0
2nd trimester	174	31.5
3rd trimester	307	55.5

Medical problem/condition		
None	342	61.8
Twin pregnancy	6	1.1
Hypertension	3	0.5
Hypertension in pregnancy	11	2.0
Type I diabetes mellitus	19	3.4
Gestational diabetes mellitus	129	23.3
Heart problem	3	0.5
Thyroid	3	0.5
Asthma	25	4.5
Others	12	2.2

**Table 2:** Knowledge on the benefits and contraindications of ANE (*n* = 553)

Variables	Number (*n*)	Frequency (%)
Ever heard about antenatal exercises		
Yes	311	56.2
No	242	43.8

What types of ANE have you heard?		
Relaxation or breathing	369	66.7
Back care exercise	175	31.6
Abdominal exercise	176	31.8
Circulatory exercise	272	49.2
Aerobic	417	75.4
Yoga	398	72.0
Cycling	438	79.2
Pelvic floor exercise	234	42.3

Benefits of ANE		
ANE can reduce back pain		
Yes	395	71.4
No	158	28.6
ANE prevents excessive weight gain during pregnancy		
Yes	361	65.3
No		34.7
Exercise strengthens pelvic floor muscle		
Yes	351	63.5
No	202	36.5
Reduces risk of gestational diabetes		
Yes	313	56.6
No	240	43.4
Exercise increases energy and stamina during pregnancy		
Yes	421	76.1
No	132	23.9
Exercise eases labour process		
Yes	447	80.8
No	106	19.2
Facilitate postnatal recovery time		
Yes	364	65.8
No	189	34.2

Contraindication of ANE		
Chest pain during pregnancy		
Yes	299	54.1
No	254	45.9
Pain on abdominal during pregnancy		
Yes	349	63.1
No	204	36.9
Back pain during pregnancy		
Yes	355	64.2
No	198	35.8
Uncontrolled DM during pregnancy		
Yes	168	30.4
No	385	69.6
Uncontrolled HPT during pregnancy		
Yes	168	30.4
No	385	69.6
Uterine contraction		
Yes	257	46.5
No	296	53.5
Vaginal bleeding during pregnancy		
Yes	275	49.7
No	278	50.3
Premature labour during pregnancy		
Yes	258	46.7
No	295	53.3
Headache/dizziness during pregnancy		
Yes	305	55.2
No	248	44.8
Decreased fetus movement		
Yes	245	44.3
No	308	55.7
Adequate knowledge	256	46.3
Inadequate knowledge	297	53.7

**Table 3:** Participant’s attitude (*n* = 553)

Variables	Number (*n*)	Frequency (%)
Antenatal exercise:		
Is essential during pregnancy		
Agree	527	95.3
Disagree	26	4.7
Will prevent pregnancy complications		
Agree	522	94.4
Disagree	31	5.6
Regularly will facilitates normal delivery		
Agree	518	93.7
Disagree	35	6.3
Will help faster recovery after delivery		
Agree	476	86.1
Disagree	77	13.9
Prescribed by HCPs are safe for your baby		
Agree	488	88.2
Disagree	65	11.8
Does not suit our culture		
Agree	66	11.9
Disagree	487	88.1
Can be performed without advice or recommendation from HCPs		
Agree	131	23.7
Disagree	422	76.3
Are safe to practice		
Agree	504	91.1
Disagree	49	8.9
Will feel energetic doing exercise		
Agree	492	89.0
Disagree	61	11.0
Have sufficient information on exercise		
Agree	230	41.6
Disagree	323	58.4
Personally, like doing exercise		
Agree	449	41.2
Disagree	104	18.8
Household activities give adequate physical exercises to pregnant women		
Agree	202	36.5
Disagree	351	63.5
Enough time to do exercise daily		
Agree	318	57.5
Disagree	235	42.5
Get enough family support for doing exercise		
Agree	376	68.0
Disagree	177	32.0

Positive attitude	455	82.3
Negative attitude	98	17.7

Note: HCPs = health care professional

**Table 4:** Participant’s practice of ANE (*n* = 553)

Variables	Number (*n*)	Frequency (%)
Practising exercise		
Yes	311	56.2
No	242	43.8

*Walking		
Not perform	34	6.1
< 3 times/week	169	30.6
> 3 times/week	350	63.3

*Circulatory exercise		
Not perform	178	32.2
< 3 times/week	198	68.0
> 3 times/week	177	32.0

*Abdominal exercise		
Not perform	312	56.4
< 3 times/week	185	33.5
> 3 times/week	56	10.1

*Pelvic floor exercise		
Not perform	292	52.8
< 3 times/week	188	34.0
> 3 times/week	73	13.2

*Breathing/relaxation exercise		
Not perform	177	32.0
< 3 times/week	192	34.7
> 3 times/week	184	33.3

Duration of exercise per session (*n* = 311)		
< 20 minutes	96	30.9
≥ 20 minutes	215	69.1

Practice level		
Good practice (> 3 times/week, minimum 20 minutes/session)	215	38.9
Poor practice (< 3 times/week, less than 20 minutes/session)	338	61.1

Note:

* Denotes that multiple answers

**Table 5 t5-12mjms3201_oa:** Factors affecting participant’s knowledge, attitude and practice of ANE

Variables	Knowledge		Attitude		Practice	
	COR (95% CI)	AOR (95% CI)	COR (95% CI)	AOR (95% CI)	COR (95% CI)	AOR (95% CI)
Educational level						
Primary	1 ref	1 ref	1 ref	1 ref	1 ref	1 ref
Secondary	0.46 (0.3–0.7)		0.36 (0.1–1.9)	0.45 (0.1–2.3)	0.69 (0.2–3.2)	0.38 (0.1–2.2)
Tertiary	0.51 (0.4–0.7) **	0.45 (0.3–0.6) **	0.97 (0.6–1.5)	1.06 (0.7–1.7)	1.02 (0.2–4.7)	0.54 (0.1–3.2)

Parity						
No children	1 ref	1 ref	1 ref	1 ref	1 ref	1 ref
1–2 children	1.45 (1.0–2.1)	1.43 (0.9–2.2)	1.14 (0.7–1.9)	1.03 (0.6–1.7)	1.06 (0.7–1.6)	0.92 (0.6–1.4)
> 3 children	1.23 (0.8–2.0)	1.4 (0.8–2.6)	1.11 (0.6–2.0)	0.098 (0.5–1.9)	1.1 (0.7–1.8)	0.99 (0.6–1.7)

Pre-pregnancy exercise						
Yes	1.20 (0.9–1.9)	0.77 (0.54–1.1)	1.87 (1.2–2.9) **	1.7 (1.1–2.7) *	2.39 (1.7–3.4) **	2.12 (1.4–3.0) **
No	1 ref	1 ref	1 ref	1 ref	1 ref	1 ref

Advice on exercise						
Yes	1.55 (1.1–2.2) **	1.55 (1.1–2.2) *	2.09 (1.3–3.3) **	1.95 (1.2–3.1) **	2.36 (1.7–3.4) **	1.97 (1.3–2.9) **
No	1 ref	1 ref	1 ref	1 ref	1 ref	1 ref

Attitude						
Positive					3.3 (2.0–5.9) **	2.98 (1.7–5.2) **
Negative					1 ref	1 ref

Notes: AOR = adjusted odd ratio; COR = crude odd ratio;

* *p* < 0.05;

** *p* < 0.01

## References

[1] Watson ED, Norris SA, Draper CE, Jones RA, van Poppel MNM, Micklesfield LK (2016). “Just because you’re pregnant, doesn’t mean you’re sick!” A qualitative study of beliefs regarding physical activity in black South African women. BMC Pregnancy Childbirth.

[2] Szumilewicz A, Wojtyła A, Aleksandra Z, Drobnik-Kozakiewicz I, Sawczyn M, Kwitniewska A (2013). Influence of prenatal physical activity on the course of labour and delivery according to the new Polish standard for perinatal care. Ann Agric Environ Med.

[3] Mbada CE, Adebayo OE, Adeyemi AB, Arije OO, Dada OO, Akinwande OA (2014). Knowledge and attitude of Nigerian pregnant women towards antenatal exercise: a cross-sectional survey. ISRN Obstet Gynecol.

[4] (2020). Physical activity and exercise during pregnancy and the postpartum period: ACOG Committee Opinion, Number 804. Obstet Gynecol.

[5] Davenport MH, Marchand A, Mottola MF, Poitras VJ, Gray CE, Jaramillo Garcia A (2019). Exercise for the prevention and treatment of low back, pelvic girdle and lumbopelvic pain during pregnancy: a systematic review and meta-analysis. Br J Sports Med.

[6] World Health Organization (2015). Trends in maternal mortality 1990 to 2015: estimates by WHO, UNICEF, UNFPA, World Bank Group and the United Nations Population Divisions. [Internet].

[7] Silva-Jose C, Sánchez-Polán M, Barakat R, Gil-Ares J, Refoyo I (2022). Level of physical activity in pregnant populations from different geographic regions: a systematic review. J Clin Med.

[8] Abdullah WH, Najib BM (2019). Knowledge and attitude of pregnant women towards antenatal exercise in Erbil City. Erbil J Nurs Midwifery.

[9] Al-Youbi GM, Elsaid T (2020). Knowledge, attitude, and practices on exercise among pregnant females attending Al-Wazarat Health Center, Riyadh, Saudi Arabia. J Fam Med Prim Care.

[10] Janakiraman B, Gebreyesus T, Yihunie M, Genet MG (2021). Knowledge, attitude, and practice of antenatal exercises among pregnant women in Ethiopia: a cross-sectional study. PLoS One.

[11] Nascimento SL, Surita FG, Godoy AC, Kasawara KT, Morais SS (2015). Physical activity patterns and factors related to exercise during pregnancy: a cross sectional study. PLoS One.

[12] Coll CVN, Domingues MR, Gonçalves H, Bertoldi AD (2017). Perceived barriers to leisure-time physical activity during pregnancy: A literature review of quantitative and qualitative evidence. J Sci Med Sport.

[13] Syed Nor SF, Idris IB, Md Isa Z (2022). Physical inactivity in early pregnancy and the determinants in an urban city setting of Kuala Lumpur, Malaysia. BMC Public Health.

[14] Mohamad Yusof NY, Mohd Zulkefli NA, Minhat HS, Ahmad N (2020). Predictors of physical inactivity among antenatal women: a systematic review. Mal J Med Health Sci.

[15] Mbada CE, Adebayo OE, Taofeek Oluwole A, Faremi FA, Oginni MO, Ogundele AO (2015). Practice and pattern of antenatal and postnatal exercise among Nigerian women: a cross-sectional study. Int J Women’s Heal Reprod Sci.

[16] Juliawati M, Rosediani M, Nik Rosmawati NH, Norwati D (2019). Pelvic floor muscle exercise education and factors associated with implementation among antenatal women in Hospital Universiti Sains Malaysia. Korean J Fam Med.

[17] Department of Statistics Malaysia (2020). Statistic of states [Internet].

[18] Chan YH (2004). Biostatistics 202: logistics regression analysis. Singapore Med J.

[19] Nkhata LA, Munalula-nkandu E, Shula H (2014). The knowledge, attitudes and practices towards exercise among women attending antenatal care at the university teaching hospital in Lusaka, Zambia. J Sci Res Rep.

[20] Wijesiriwardana WS, Gunawardena NS (2015). Knowledge, attitudes and practices regarding antenatal exercises among pregnant mothers attending De Soyza Maternity Hospital Colombo. Sri Lanka J Obstet Gynaecol.

[21] Sitot A, Workye H (2022). Assessment of knowledge, attitude and practice towards ante natal exercise among pregnant women attending antenatal care at Health centers of Mekelle, Tigray Region, Ethiopia, 2020. PLoS One.

[22] Bayisa D, Waltengus F, Lake S, Wakuma B, Bayisa L, Chala M (2022). Pregnant women’s knowledge, attitudes, and associated factors toward physical exercise during pregnancy among those attending antenatal care at Bahir Dar city, Northwest Ethiopia. SAGE Open Med.

[23] Sujindra E, Bupathy A, Suganya A, Praveena R (2015). Knowledge, attitude, and practice of exercise during pregnancy among antenatal mothers. Int J Educ Psychol Res.

[24] Ribeiro CP, Milanez H (2011). Knowledge, attitude and practice of women in Campinas, São Paulo, Brazil with respect to physical exercise in pregnancy: a descriptive study. Reprod Health.

[25] Lapointe L, Ramaprasad J, Vedel I (2014). Creating health awareness: a social media enabled collaboration. Health Technol.

[26] Lee R, Thain S, Tan LK, Teo T, Tan KH (2021). Asia-Pacific consensus on physical activity and exercise in pregnancy and the postpartum period. BMJ Open Sport Exerc Med.

[27] Connolly CP, Feltz DL, Pivarnik JM (2014). Overcoming barriers to physical activity during pregnancy and the postpartum period: the potential impact of social support. Kinesiol Rev.

[28] Hashmi M, Ain QU, Shaikh NUS, Valecha J (2020). Knowledge and attitude of pregnant women regarding antenatal exercises. J Pharm Res Int.

